# Supplementary material for: Patterns of Intron Gain and Loss in Fungi
Source: PLoS Biol. 2004 Nov 30;2(12):e422. doi: 10.1371/journal.pbio.0020422 (PMC532390; doi:10.1371/journal.pbio.0020422)
Supplement: Table S1 — Also available at http://genes.mit.edu/NielsenEtAl/. (4.3 MB ZIP). [file pbio.0020422.st001.zip › NielsenEtAl/html/180.html]

AN1780.1.NCU05971.1.MG00596.1.FG09309.1


```
 CLUSTAL W (1.82) Multiple Sequence Alignments - Introns Inserted


Sequence 1: MG00596.1	451 aa
Sequence 2: FG09309.1	499 aa
Sequence 3: NCU05971.1	451 aa
Sequence 4: AN1780.1	506 aa
Alignment Length: 523 aa
Number Identitical Residues: 181 aa
Alignment Score (without introns) 9768


MG00596.1 	--MPFSPVRQVG------------------------------------A1S~SA1SELQF
NCU05971.1	-----------------------------------------------MI~I1SA~ADLEF
FG09309.1 	MRLPRAPFRSVDPRLPLRAPSTRKISTISLRARPTLPSNIPARLRTYAS~V~SA~SELRF
AN1780.1  	MVACRSLVRQWPRYLRHPSSLRALSRLSQPRVQTQFVR------RGYAS~I~SA~AELKF
          	     :   .        :.        .    .                  ** ::*.*

MG00596.1 	GQPVHETHPHILKAGEL1TPGISAQEYYDRRQNLAALLPENGVAILVSAELRYRSGAVFF
NCU05971.1	GQPVYETHPHILQPGEL~TPGITAQEYHERRSKLAFALPENGIAILASSELKYRSGAVFF
FG09309.1 	GQPVHETHPHILKAGEI~TQGITAQEYADRRAALAHSMTDGSVAVLHAASLQYKSGAVFH
AN1780.1  	GQPLHETHPHLLDPGEL1TPGITALEYAHRRSRLANKLPKHAIAVLAAAEVTYRASGIFN
          	***::*****:*..**: * **:* ** .**  **  :.. .:*:* ::.: *::..:* 

MG00596.1 	PFRQESNFLYLTG~FAEQDAVAVIR~KTG--QHDHEFRLYVRPKDASAEQWSGPWSGVDA
NCU05971.1	PFRQDSNFLYLTG~FSEPDSLAIIQ~KTGPKPGDYVFHLFCRPKDPRAEQWSGPWSGLQA
FG09309.1 	AYRQESNFLYLTG~WMEGDAIAVIQ~KTGPNYGDYIFHMFVKPKNAREEQWNGYRNGVDA
AN1780.1  	VFRQDSNFFYLTG1FNEPDALAIII1NDG-SGDNHLFHLYCREKDAKAELWDGARSGTQA
          	 :**:***:**** : * *::*:*  : * .  :: *::: : *:.  * *.*  .* :*

MG00596.1 	AMDVWNADV~AGPISAAAHEVGQLAKAASPAIYTDAPAYGGGTGAVK-------------
NCU05971.1	AEDVFNADF~TGDIAKVETLLPPILRGAG-KVYTDITPSTPVTGQLVTTLLS--------
FG09309.1 	ARDIFNADE~AHSIYEAEHLLPKVLDRAK-LIYADSPPSKGGIAATATWLWRLI------
AN1780.1  	AMDVFNADE0SGDIERISDILPKVLADAT-EIYTDIPAFNPGRSSLHRFLYGPTGTSEKL
          	* *::***  :  *      :  :   *   :*:* ..     .          .::.. 

MG00596.1 	----------PLAPLVNSLRAIKSPAEVANMRHAGRVSGRAFTAAMRGDASGSDWKYERD
NCU05971.1	---HLHLPPAPLAGLVHSLRAIKSPAEISNMRHAGRVSGRALTSAMR-----RSWSSEKD
FG09309.1 	SGKDTRPTRTPLYSVMNKLRVVKSEAEVANMRLAGQISGRVITDAMR-----QPWKREKD
AN1780.1  	KKLVDHSKVKPLRPILNEMRAFKSEEEVVQLRRVGQASGRAFTEAMR-----HSFTGEKD
          	.         **  :::.:*..**  *: ::* .*: ***.:* ***       :. *:*

MG00596.1 	LALFFDHTFSTTGCDGQAYVPVVAGGS~RGSMIHYVHNNRELP~TGEMVLVDAGG~EYGT
NCU05971.1	LEAYLNYAFTSHGLSGPAYVPVIAGGS~RGNMIHYVHNNRLLN~QDDMVLVDAGG~EYGT
FG09309.1 	LHWFLDYNFAANGCEGPAYIPVVAGGE~RANCIHYTVNNSTFD~EDEFILIDAGG~EYGT
AN1780.1  	LSSFLEYNFKLNGCDGSAFVPVVAAGS0NALSIHYTKNDDIFK2DGDMVLVDGGG0ELGT
          	*  :::: *   * .* *::**:*.*. ..  ***. *:  :   .:::*:*.** * **

MG00596.1 	YITDITRTWPINGKFTPAQRDLYEAVLKVQRSAVSLCRADSGFSLDKIHMIAQDGLRDQL
NCU05971.1	YITDITRTWPVNGKFSAPQRDLYEAVLTVQRKMVSLCRENATLSLDQIHRATEAGLREQL
FG09309.1 	YITDISRTWPASGRFTPAQRDLYQAVLNVQRSSVSLCRENSKLSLEDIHNVTARGLVDQL
AN1780.1  	YISDITRTWPVNGKFSDPQRDLYNAVLNVHRSCVSLCRESAGLSLDRLHGIAENGLKDQL
          	**:**:**** .*:*: .*****:***.*:*. ***** .: :**: :*  :  ** :**

MG00596.1 	IQLGFEFDGR~NGRAIQTLFPHHLSHYVGLDVHDTPGYSRSVPLQQGHCVTVEP~GIYVP
NCU05971.1	TLLGFDLSSG~GGGRMDVLFPHHVGHYVGLDVHDTPGYSRSLTLRQGHAVTIEP~GVYVP
FG09309.1 	RAIGFEVTMA~N---IDQLFPHHVGHYIGLDVHDCPGYSRRETLRRGHCVTIEP~GVYVP
AN1780.1  	TQLGFDLSGD0A---LRTLFPHHLGHYIGLDVHDCAGYSRGYDLKAGQCITIEP2GIYVP
          	  :**:.        :  *****:.**:****** .****   *: *:.:*:** *:***

MG00596.1 	-DDERWPEPFRGMAIRIEDSICVLDDVPLVLTSEAVKE~VA--------DVEALRA
NCU05971.1	QDDDRFPEHFRGLAVRIEDSVVVDVESPLILTTEAVKE~VV--------DIEALRE
FG09309.1 	-HDDRWPAAFRGMGVRIEDSVCVDHDSPYILSTEAVKE~VD--------DIEALRS
AN1780.1  	-DDDRWPAKFRGVGIRIEDSVCVGDDNPIVLTTEAVKE0VLTWCNSQVDDIEALRD
          	 .*:*:*  ***:.:*****: *  : * :*::***** * : ..:. .*:****
```
